# Suprainguinal Fascia Iliaca Block and Pericapsular Nerve Group Block Catheter Procedure for Postoperative Joint Motion Limitation Rehabilitation

**DOI:** 10.7759/cureus.76935

**Published:** 2025-01-05

**Authors:** Öztürk Taşkın, Ufuk Demir, Ayşe Yılmaz, Büşra Tanyıldızı Küçük, Emir Kütük, Zahide Doğanay

**Affiliations:** 1 Anesthesiology and Reanimation, Kastamonu University Faculty of Medicine, Kastamonu, TUR; 2 Orthopaedics, Kastamonu Training and Research Hospital, Kastamonu, TUR

**Keywords:** block catheter, pericapsular nerve group block, pericapsular nerve group block catheter, regional anesthesia, rehabilitation, suprainguinal approach, suprainguinal fascia iliaca block, suprainguinal fascia iliaca block catheter

## Abstract

A distal femur fracture is an orthopedic condition that can be seen in all age groups and causes severe pain both preoperatively and postoperatively. Poorly managed pain can lead to hemodynamic changes, the need for additional analgesic drugs containing opioids, increased hospital stay, delayed mobilization, and patient dissatisfaction. Regional anesthesia techniques can be used for preoperative and postoperative analgesia. Suprainguinal fascia iliaca block and pericapsular nerve group block are applications included in regional techniques that preserve motor functions of the thigh. We also share our experience of suprainguinal fascia iliaca block and pericapsular nerve group block catheter application that we applied to a patient who underwent tenolysis surgery due to a distal femur fracture and adhesion in the quadriceps muscle group.

## Introduction

Distal femur fractures account for 3-6% of all femur fractures and usually occur in a bimodal distribution resulting from high-energy trauma in young patients or low-energy injuries in elderly osteoporotic patients [1,2]. Severe pain can lead to an increased stress response and dramatic hemodynamic changes, which can then trigger serious cardiovascular and cerebrovascular complications such as cerebral hemorrhage and myocardial infarction and develop into chronic pain. Various regional anesthesia techniques have been described to provide postoperative analgesia in these patients. Regional anesthesia techniques can be used not only for postoperative analgesia but also for chronic pain [3]. Fascia iliaca compartment block (FICB) and pericapsular nerve group (PENG) block are applications included in these techniques that preserve thigh motor functions.

Nerve blocks have been shown to effectively reduce pain from hip and knee operations and provide rapid-onset analgesia that is more effective than traditional analgesia. FICB can simultaneously block the femoral nerve, obturator nerve, and lateral femoral cutaneous nerve to achieve satisfactory analgesia in patients with hip fractures [4-6]. In recent years, a new approach to perform suprainguinal fascia iliaca block (SIFIB) has been introduced in the clinic. This technique uses the anterior superior iliac crest as a bony landmark to identify the iliac fascia and iliac muscle [7]. The PENG block is a new regional analgesia technique that can reduce pain after hip surgery and fractures, providing better analgesia than other peripheral blocks used in these procedures. The PENG block is typically used to provide analgesia after hip or thigh injuries or surgeries, such as acetabular fractures, femoral neck or midshaft fractures, hip replacement, hip arthroscopy, and knee surgery [8]. A recent study demonstrated effective surgical anesthesia with PENG block for a medial thigh lesion [8]. These two techniques can be used as single shots or can provide continuous analgesia with an inserted catheter [9].

In our literature search, we found that there were no publications on the SIFIB catheter and the number of PENG catheters was limited. For this reason, we wanted to share with you our SIFIB and PENG catheter applications that we applied intermittently in a patient.

## Case presentation

**Figure 1:**
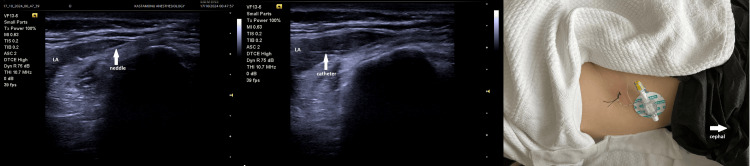
Suprainguinal fascia iliaca block catheter LA: local anesthetic solution

**Figure 2:**
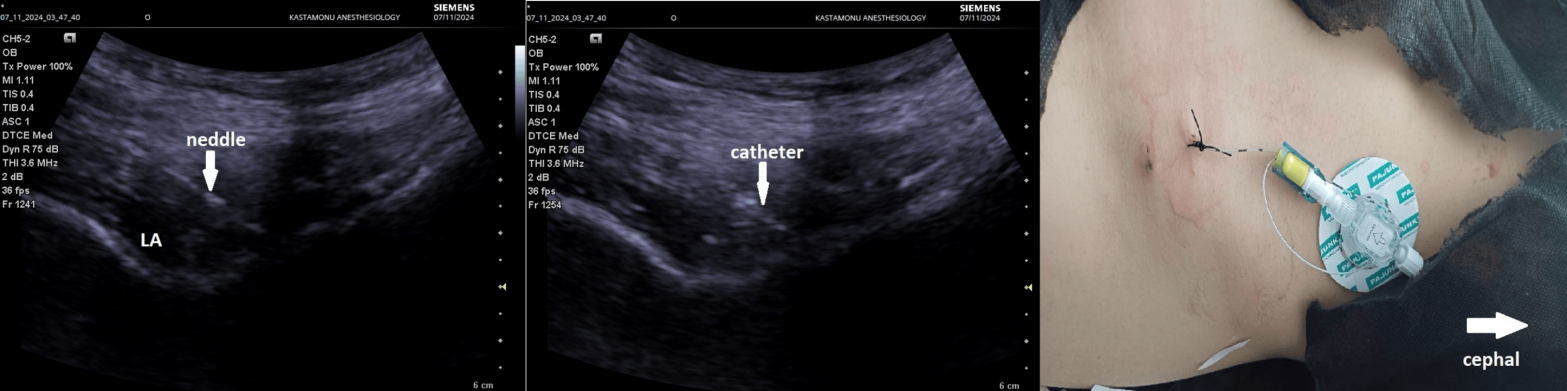
Pericapsular nerve group block catheter LA: local anesthetic solution

A 23-year-old, American Society of Anesthesiologists (ASA) II (anemia), 70 kg, 165 cm tall (body mass index, 25.7) female patient underwent tenolysis surgery due to a distal femur fracture and subsequent adhesion in the quadriceps muscle group after falling from a height by spinal anesthesia two months ago. The patient developed pain in the knee area and limited knee joint flexion after the surgery. She consulted with a physical therapy and rehabilitation physician and was advised to exercise with a continuous passive motion (CPM) device for half an hour, three times a day for two weeks. The visual analog scale (VAS) value was 7 at rest and 9 during movement. The patient's range of motion was observed as 30°. SIFIB catheter application was planned for the patient. The procedure was explained to the patient and consent was obtained. SIFIB block was performed with a mixture of 0.5% bupivacaine (20 ml) and saline (30 ml) under ultrasound (USG) guidance, and then the block catheter was placed (Figure 1). The catheter position was confirmed with 2 cc saline. For multimodal analgesia, the patient was given 3×1 g paracetamol and 2×1 g dexketoprofen. The patient's VAS scores (1-8-16-24 hours) and maximum knee flexion angles during exercise, whether there was a motor block, and whether there was additional analgesia were recorded for five days. While the patient's first-, eighth-, and 16th-hour VAS values ​​were evaluated with the CPM device during movement, the 24th-hour VAS value was evaluated during the patient's active movement. The VAS score was evaluated at every 24th hour; after evaluation, the same block dose was repeated routinely. After the block was performed on the catheter on the fifth day, it was withdrawn due to the risk of catheter infection. The patient was consulted again by us 24 hours later. During the treatment with the CPM device, it was observed that the VAS value was 6 at rest and 8 with exercise and the range of motion of the joint decreased accordingly (80°). A PENG block catheter was planned to be applied to the patient. The block was performed with a mixture of bupivacaine 0.5% (15 ml) and lidocaine 2% (5 ml) under USG guidance, and then the catheter was placed (Figure 2). The catheter position was confirmed with 2 cc saline. The multimodal analgesia management applied after the SIFIB catheter was applied in the same way, and the same parameters were recorded for five days. The PENG block catheter was removed at the end of the fifth day.

In both approaches, pain scores were lower both at rest and during exercise. There was no need for opioids or additional analgesia and no motor blockade developed. The patient's maximum flexion angles during exercise increased each day (Table 1). No complications developed in either application.

**Table 1:** Parameter values ​​after SIFIB and PENG block catheter SIFIB: suprainguinal fascia iliaca block; PENG: pericapsular nerve group; VAS: visual analog scale

Block type	Parameters		Before block	After block																			
				Day 1				Day 2				Day 3				Day 4				Day 5			
		Hour	0	1	8	16	24	1	8	16	24	1	8	16	24	1	8	16	24	1	8	16	24
SIFIB	VAS	Rest	7	1	0	2	4	0	0	4	4	3	0	0	4	0	0	0	2	0	0	2	2
		Movement	9	4	4	4	4	4	4	4	4	4	4	4	4	4	3	3	4	4	2	4	4
PENG	VAS	Rest	6	1	0	1	3	0	1	1	2	0	0	1	2	0	0	1	1	0	0	1	2
		Movement	8	3	3	3	4	3	3	4	4	3	3	4	4	2	2	2	3	2	2	2	4
SIFIB	Angle (maximum)		30°	50°				80°				100°				110°				120°			
	Motor block		None	None				None				None				None				None			
	Rescue analgesic			None				None				None				None				None			
PENG	Angle (maximum)		80°	90°				100°				105°				115°				120°			
	Motor block		None	None				None				None				None				None			
	Rescue analgesic			None				None				None				None				None			

## Discussion

Studies have reported that both SIFIB and PENG block are good options for postoperative analgesia in hip, knee, and medial thigh region operations [7]. A review by Scurrah et al. emphasized that nerve blocks such as SIFIB should be routinely added to multimodal pain management for hip fracture patients. In the same article, they noted that the block effectively reduced opioid requirements, decreased the incidence of delirium, and had a positive effect on morbidity, mortality, and quality of life [4]. In the cadaver study conducted by Hebbard et al., dye distribution was examined with SIFIB catheter application, and they emphasized that both femoral and lateral femoral cutaneous nerves were stained. In addition, in the same study, it was mentioned that the SIFIB catheter application is actively used in the clinic [10]. Girón-Arango et al. suggested that the PENG block targets the joint branches of the femoral and accessory obturator nerve [11]. Pahwa et al. stated that VAS scores decreased in a patient with a femoral neck fracture after PENG block catheter application to relieve pain before surgery [12]. In an article published by Guay et al., they reported that peripheral nerve blocks for hip fractures reduced pain within 30 minutes and shortened the initial mobilization time [5]. In our case, after SIFIB and PENG block catheter applications, lower VAS scores, higher maximum joint flexion angles during exercise, and the need for no opioid-containing analgesic agents support this.

Pain scores reported in both block applications indicate that the blocks are effective in controlling postoperative pain, especially with consistently low VAS scores at rest. The ability of both blocks to maintain these scores with minimal fluctuations is remarkable and suggests that the block may provide continuous analgesic coverage with catheter placement. Low pain scores, especially during exercise, indicate that these two blocks can contribute significantly to patient comfort, which is very important for early postoperative mobilization and rehabilitation. In patients whose treatment and mobilization process will take a long time, the application of these two block catheters will provide better and longer-term pain management, long-term comfort, and, as a result, early mobilization and shorter hospital stay. The absence of opioid use in the catheterized patient during this period is also more advantageous in terms of addiction risks and side effects associated with these drugs.

## Conclusions

SIFIB and PENG block catheters offer a promising alternative with a favorable safety profile and significant analgesic efficacy for pain management after hip or thigh injuries or surgeries such as hip acetabular fractures, femoral neck or midshaft fractures, hip replacement, hip arthroscopy, and knee surgery. The use of these blocks in multimodal analgesia management has positive effects on opioid requirement and dependence, morbidity-mortality, and quality of life. These blocks also shorten the length of hospital stay, reduce costs, and increase patient satisfaction. However, further research is needed to determine their role in the broader context of orthopedic postoperative pain management.
